# The SNP rs6500843 in 16p13.3 is associated with survival specifically among chemotherapy-treated breast cancer patients

**DOI:** 10.18632/oncotarget.3506

**Published:** 2015-03-10

**Authors:** Rainer Fagerholm, Marjanka K. Schmidt, Sofia Khan, Sajjad Rafiq, William Tapper, Kristiina Aittomäki, Dario Greco, Tuomas Heikkinen, Taru A. Muranen, Peter A. Fasching, Wolfgang Janni, Richard Weinshilboum, Christian R. Loehberg, John L. Hopper, Melissa C. Southey, Renske Keeman, Annika Lindblom, Sara Margolin, Arto Mannermaa, Vesa Kataja, Georgia Chenevix-Trench, kConFab Investigators, Diether Lambrechts, Hans Wildiers, Jenny Chang-Claude, Petra Seibold, Fergus J. Couch, Janet E. Olson, Irene L. Andrulis, Julia A. Knight, Montserrat García-Closas, Jonine Figueroa, Maartje J. Hooning, Agnes Jager, Mitul Shah, Barbara J. Perkins, Robert Luben, Ute Hamann, Maria Kabisch, Kamila Czene, Per Hall, Douglas F. Easton, Paul D.P. Pharoah, Jianjun Liu, Diana Eccles, Carl Blomqvist, Heli Nevanlinna

**Affiliations:** ^1^ Department of Obstetrics and Gynecology, University of Helsinki and Helsinki University Hospital, Helsinki, Finland; ^2^ Netherlands Cancer Institute, Antoni van Leeuwenhoek hospital, Amsterdam, The Netherlands; ^3^ Faculty of Medicine, University of Southampton, Southampton General Hospital, Southampton, UK; ^4^ Department of Clinical Genetics, University of Helsinki and Helsinki University Hospital, Helsinki, Finland; ^5^ Department of Gynecology and Obstetrics, University Hospital Erlangen, Friedrich-Alexander University Erlangen-Nuremberg, Erlangen, Germany; ^6^ Department of Medicine, Division of Hematology and Oncology, University of California at Los Angeles, Los Angeles, CA, USA; ^7^ Department of Gynecology and Obstetrics, University Hospital Ulm, Ulm, Germany; ^8^ Division of Clinical Pharmacology, Department of Molecular Pharmacology and Experimental Therapeutics, Mayo Clinic College of Medicine, Mayo Medical School-Mayo Foundation, Rochester, MN, USA; ^9^ University Breast Center Franconia, Department of Gynecology and Obstetrics, University Hospital Erlangen, Friedrich-Alexander University Erlangen-Nuremberg, Comprehensive Cancer Center Erlangen-EMN, Erlangen, Germany; ^10^ Centre for Epidemiology and Biostatistics, Melbourne School of Population and Global Health, The University of Melbourne, Melbourne, Victoria, Australia; ^11^ Department of Pathology, The University of Melbourne, Melbourne, Victoria, Australia; ^12^ Department of Molecular Medicine and Surgery, Karolinska Institutet, Stockholm, Sweden; ^13^ Department of Oncology - Pathology, Karolinska Institutet, Stockholm, Sweden; ^14^ School of Medicine, Institute of Clinical Medicine, Pathology and Forensic Medicine, University of Eastern Finland, Kuopio, Finland; ^15^ Cancer Center of Eastern Finland, University of Eastern Finland, Kuopio, Finland; ^16^ Imaging Center, Department of Clinical Pathology, Kuopio University Hospital, Kuopio, Finland; ^17^ Cancer Center, Kuopio University Hospital, Kuopio, Finland; ^18^ Department of Genetics, QIMR Berghofer Medical Research Institute, Brisbane, Australia; ^19^ Peter MacCallum Cancer Center, Melbourne, Victoria, Australia; ^20^ Vesalius Research Center (VRC), VIB, Leuven, Belgium; ^21^ Laboratory for Translational Genetics, Department of Oncology, University of Leuven, Leuven, Belgium; ^22^ Multidisciplinary Breast Center, Medical Oncology, University Hospital Leuven, Leuven, Belgium; ^23^ Division of Cancer Epidemiology, German Cancer Research Center (DKFZ), Heidelberg, Germany; ^24^ Department of Laboratory Medicine and Pathology, Mayo Clinic, Rochester, MN, USA; ^25^ Department of Health Sciences Research, Mayo Clinic, Rochester, MN, USA; ^26^ Lunenfeld-Tanenbaum Research Institute of Mount Sinai Hospital, Toronto, ON, Canada; ^27^ Department of Molecular Genetics, University of Toronto, Toronto, ON, Canada; ^28^ Prosserman Centre for Health Research, Lunenfeld-Tanenbaum Research Institute of Mount Sinai Hospital, Toronto, ON, Canada; ^29^ Division of Epidemiology, Dalla Lana School of Public Health, University of Toronto, Toronto, ON, Canada; ^30^ Division of Genetics and Epidemiology, Institute of Cancer Research, Sutton, UK; ^31^ Breakthrough Breast Cancer Research Centre, Division of Breast Cancer Research, The Institute of Cancer Research, London, UK; ^32^ Division of Cancer Epidemiology and Genetics, National Cancer Institute, Rockville, MD, USA; ^33^ Department of Medical Oncology, Erasmus MC Cancer Institute, 3008 AE Rotterdam, The Netherlands; ^34^ Centre for Cancer Genetic Epidemiology, Department of Oncology, University of Cambridge, UK; ^35^ Clinical Gerontology, Department of Public Health and Primary Care, University of Cambridge, Cambridge, UK; ^36^ Molecular Genetics of Breast Cancer, German Cancer Research Center (DKFZ), Heidelberg, Germany; ^37^ Department of Medical Epidemiology and Biostatistics, Karolinska Institutet, Stockholm, Sweden; ^38^ Centre for Cancer Genetic Epidemiology, Department of Public Health and Primary Care, University of Cambridge, Cambridge, UK; ^39^ Centre for Cancer Genetic Epidemiology, Department of Oncology, University of Cambridge, Cambridge, UK; ^40^ Human Genetics Division, Genome Institute of Singapore, Singapore; ^41^ Department of Oncology, University of Helsinki and Helsinki University Hospital, Helsinki, HUS, Finland

**Keywords:** breast cancer, survival, SNP, chemotherapy, cell cycle

## Abstract

We have utilized a two-stage study design to search for SNPs associated with the survival of breast cancer patients treated with adjuvant chemotherapy. Our initial GWS data set consisted of 805 Finnish breast cancer cases (360 treated with adjuvant chemotherapy). The top 39 SNPs from this stage were analyzed in three independent data sets: iCOGS (n=6720 chemotherapy-treated cases), SUCCESS-A (n=3596), and POSH (n=518). Two SNPs were successfully validated: rs6500843 (any chemotherapy; per-allele HR 1.16, 95% C.I. 1.08-1.26, p=0.0001, p_(adjusted)_=0.0091), and rs11155012 (anthracycline therapy; per-allele HR 1.21, 95% C.I. 1.08-1.35, p=0.0010, p_(adjusted)_=0.0270). The SNP rs6500843 was found to specifically interact with adjuvant chemotherapy, independently of standard prognostic markers (p_(interaction)_=0.0009), with the rs6500843-GG genotype corresponding to the highest hazard among chemotherapy-treated cases (HR 1.47, 95% C.I. 1.20-1.80). Upon *trans*-eQTL analysis of public microarray data, the rs6500843 locus was found to associate with the expression of a group of genes involved in cell cycle control, notably *AURKA,* the expression of which also exhibited differential prognostic value between chemotherapy-treated and untreated cases in our analysis of microarray data. Based on previously published information, we propose that the eQTL genes may be connected to the rs6500843 locus via a RBFOX1-FOXM1-mediated regulatory pathway.

## INTRODUCTION

Breast cancer is the most common type of malignancy and one of the leading causes of death among women worldwide. Susceptibility to the disease is heavily influenced by inherited factors, with roughly 50% of familial breast cancer risk attributable to genetic variation [1, 2]. Genetic variation also contributes to the phenotypic spectrum of the disease, with both high-penetrance and common variants associating with various histopathological features, most notably estrogen receptor status [3].

The impact of genetic variation on breast cancer prognosis is less well understood. The prognosis and indicated treatment for breast cancer is influenced by tumor grade, stage, HER2 expression, and hormone receptor status [4, 5], and it is plausible that genetic variants associated with these features would be of prognostic and predictive interest. Additionally, genetic variation may contribute to breast cancer survival independently of these markers, potentially by affecting the efficacy of the treatment. For example, prognostic and predictive SNPs have been reported in the *TP53* gene and its regulatory network, as well as in genes involved in oxidative stress [6-10]. Such findings have emerged primarily from candidate gene based approaches, as more comprehensive GWS-based survival analyses tend to be problematic due to issues of statistical power: very large sample sizes are required to reach GWS significance. The collaborative iCOGS genotyping project [2] now enables this type of a study, with over 30,000 genotyped breast cancer cases eligible for survival analysis, of which 17828 cases have adjuvant treatment information available, although it can still be challenging to detect modest effect sizes in smaller subgroups, such as genetic effects that modulate survival after a specific type of adjuvant treatment.

We have utilized a two-stage study design to search for genetic variants associated with survival after adjuvant chemotherapy in breast cancer. First, we conducted an initial pilot GWS in an event-enriched set of 805 Finnish breast cancer cases. We then sought to validate our findings in an independent validation material consisting of three separate studies: iCOGS [2], POSH [11], and SUCCESS-A (dbGaP Study Accession: phs000547.v1.p1). SNPs associated with survival after treatment were further characterized using eQTL analysis in two large gene expression data sets and subsequent *in silico* analyses.

## RESULTS

### rs6500843 and rs11155012 are associated with survival after adjuvant chemotherapy

Initially, a genome-wide study (Stage I: HEBCS-GWS; n = 805) was conducted to discover candidate SNPs that may be associated with breast cancer survival, with an emphasis on treatment-based subgroups. This initial stage of the analysis was carried out using three different endpoints in parallel: five-year BDDM (breast cancer death or distant metastasis), 10-year breast cancer specific survival, and 10-year overall survival, using a codominant genetic model (test for heterogeneity between genotypes). We used fairly lenient statistical criteria to select candidate SNPs at this stage: a SNP would be selected for validation if it fulfilled any of the following criteria: 1) main effect p < 10^−4^ and p < 0.01 among chemotherapy-treated cases, 2) p < 10^−4^ among chemotherapy-treated cases, or 3) p < 10^−3^ among chemotherapy-treated cases and a homozygote-associated hazard ratio > 3.0. In total, we identified 45 putative hits from this Stage I pilot that were represented by 39 nominal and tagging SNPs on the iCOGS chip (Supplementary Table 1).

The candidate SNPs from Stage I were then analyzed in Stage II, comprising of the iCOGS, POSH and SUCCESS-A data sets. All Stage II survival analyses were restricted to cases who had received adjuvant chemotherapy. The analyses were run under the additive genetic model with left-truncated follow-up times, adjusted for patient age at diagnosis, and (in the case of iCOGS) stratified by study. Ten-year overall survival (death from any cause) was used as the end-point in these analyses for reasons of data availability and consistency. Three of the SNPs were statistically significant at this stage (Benjamini-Hochberg-adjusted p < 0.05): rs6500843 (any chemotherapy; HR 1.16, 95% C.I. 1.08 - 1.26, p = 0.0001, p_(adjusted)_ = 0.0091); rs4502225 (any chemotherapy; HR 0.78, 95% C.I. 0.67 - 0.90, p = 0.0007, p_(adjusted)_ = 0.0263); and rs11155012 (anthracycline therapy; HR 1.21, 95% C.I. 1.08 - 1.35, p = 0.0010, p_(adjusted)_ = 0.0270)(Table 1). Of these, the calculated hazard ratio for rs4502225 was in the opposite direction than in Stage I, and therefore this SNP cannot be considered a validated hit.

**Table 1:** Survival statistics for the three SNPs associated with 10-year overall survival in the Stage II analysis

		rs6500843	rs4502225	rs11155012
Chromosome		16	16	6
Position		6 870 855	78 424 831	139 151 784
Genes in LD region		*RBFOX1*	*WWOX*	*ECT2L, CCDC28A*
				
Subgroup		Chemotherapy+	Chemotherapy+	Anthracycline+
Model		additive	additive	additive
Stage I (HEBCS-GWS)				
	p-value	0.0081	0.0045	0.0073
	HR	1.36	1.55	1.50
	95% C.I.	1.08-1.70	1.14-2.09	1.12-2.02
				
Stage II (iCOGS, POSH, SUCCESS-A)				
	p-value	0.0001	0.0007	0.0010
	(Adjusted)*	0.0091	0.0263	0.0270
	HR	1.16	0.78	1.21
	95% C.I.	1.08-1.26	0.67-0.90	1.08-1.35
				
Meta-analysis (Stage I, Stage II)				
	p-value	6.96 × 10^−6^	0.0594	8.41 × 10^−5^
	HR	1.18	0.88	1.23
	95% C.I.	1.10-1.27	0.77-1.01	1.11-1.37
				

* Benjamini-Hochberg correction for multiple testing

**Figure 1:**
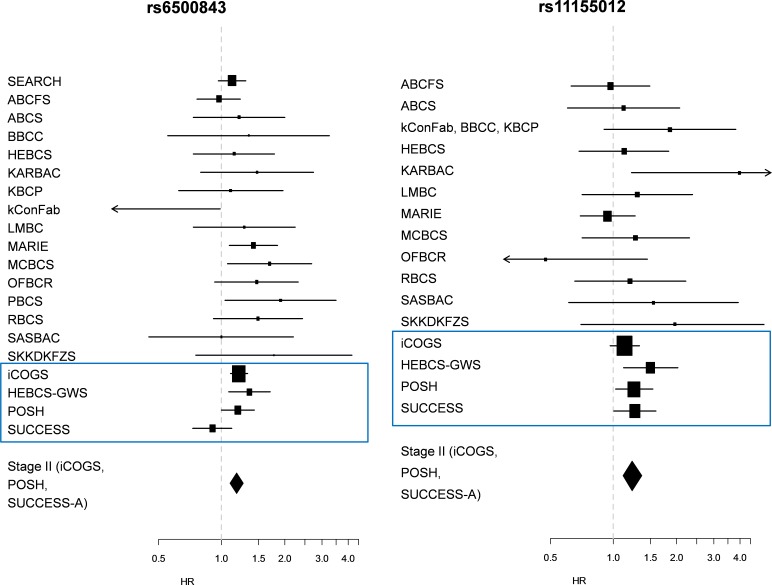
Forest plots depicting study-wise hazard ratios for the statistically significant SNPs detected in Stage II a) Hazard ratios for rs6500843 among cases treated with any adjuvant chemotherapy (additive model); b) Hazard ratios for rs11155012 among anthracycline-treated cases (additive model).

Finally, we performed a meta-analysis of both the Stage I and Stage II data sets, resulting in very slight improvement in the precision of the per-allele HR estimates for the remaining two SNPs: HR 1.18 (95% C.I. 1.10 – 1.27; p = 6.96 × 10^−6^) for rs6500843 in the chemotherapy-treated group, and HR 1.23 (95% C.I. 1.11 – 1.37; p = 8.41 × 10^−5^) for rs11155012 in the anthracycline-treated group. See Figure 1 for forest plots displaying hazard ratios from Stage I and Stage II data sets, as well as from individual participant studies within the iCOGS data set. We detected nominally significant heterogeneity (p = 0.048) among the data sets in the rs6500843 analysis, indicating some disagreement between data sets; the SNP-associated increased hazard was not seen in the SUCCESS-A data set. No heterogeneity was observed in the case of rs11155012 (p = 0.415). Neither rs6500843 nor rs11155012 were associated with tumor histopathological characteristics (Supplementary Table 2), nor were the survival analyses described above adjusted for patient or tumor characteristics except age at diagnosis. More detailed phenotypes were taken into account in the subsequent interaction analyses (see below).

### rs6500843 interacts with adjuvant chemotherapy in multivariate survival analysis

**Figure 2:**
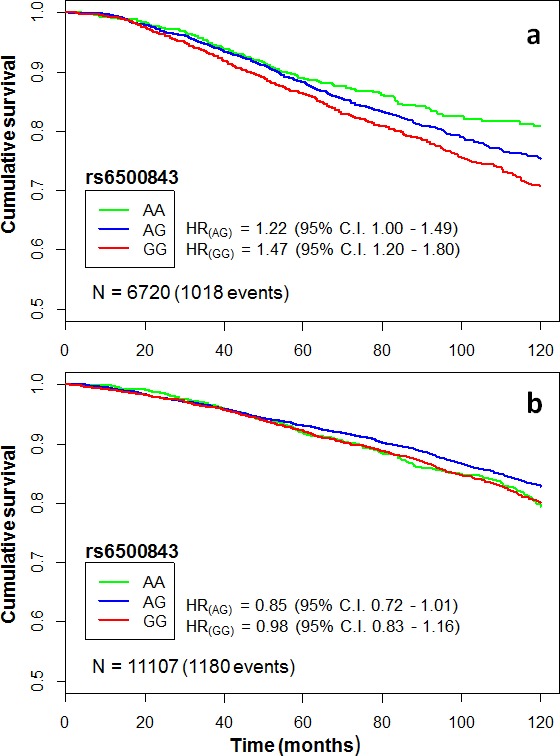
Kaplan-Meier curves illustrating cumulative 10-year overall survival among cases of the pooled iCOGS data set categorized by rs6500843 genotype The HRs indicate genotype-specific hazard ratios relative to the reference genotype (AA). Censoring marks have been omitted to make the curves clearer. The data set has been subgrouped according to treatment: a) patients treated with any adjuvant chemotherapy, b) patients who did not receive adjuvant chemotherapy.

Next, we investigated whether the observed survival effects are specific to the chemotherapy-treated subgroup, and whether these effects occur independently of standard prognostic factors. To this end, we tested for interaction between treatment and the rs6500843 and rs11155012 SNP genotypes in multivariate Cox proportional hazards models adjusted for ER, grade, tumor size, and nodal metastasis. This analysis was only performed in the iCOGS data set, as it contains both treated and non-treated cases in abundant numbers, enabling robust interaction testing. A likelihood-ratio test comparing models with interaction terms with models with only independent covariates indicated an interactive effect between rs6500843 and adjuvant chemotherapy (p_(interaction)_ = 0.0009, N = 9680 [1176 events]; Table 2), consistent with the finding that the SNP was associated with survival in the “any chemotherapy” subset in univariate analysis. See Figure 2 for a visualization of rs6500843-associated survival within the chemotherapy-treated and untreated subgroups in the iCOGS data set. Consistent with the additive model employed in the survival analyses, the genotype-specific hazard ratios for rs6500843 in the chemotherapy-treated group increase in an allele dose dependent manner (HR_(AG)_ = 1.22 (95% C.I. 1.00 - 1.49); HR_(GG)_ = 1.47 (95% C.I. 1.20 - 1.80). In contrast, no heterogeneity between genotypes can be observed in the untreated group. The conclusion of the interaction analysis did not change when radiotherapy, surgery, and adjuvant endocrine therapy were added as additional covariates in the model (p_(interaction)_ = 0.0035, N = 8943 [1110 events]). Despite its association with survival in the anthracycline-treated and any chemotherapy treated subsets, no evidence of interaction was observed for the rs11155012 SNP (Supplementary Table 3), suggesting that its possible association with survival is not entirely treatment-specific.

**Table 2:** Multivariate Cox proportional hazards model constructed to detect interaction between the SNP rs6500843 and treatment (any adjuvant chemotherapy

Model without interaction			
	HR	95% C.I.	p
rs6500843	1.05	0.97 - 1.14	0.240
Chemotherapy	1.16	0.99 - 1.35	0.066
Age	1.04	1.04 - 1.05	< 10^−16^
Grade	1.40	1.27 - 1.54	8.5 × 10^−12^
ER	0.75	0.64 - 0.86	8.1 × 10^−5^
T	1.53	1.39 - 1.69	< 10^−16^
N	2.03	1.78 - 2.31	< 10^−16^
*(Likelihood ratio test=640 on 10 df, p=0 n= 9680, number of events= 1176)*			

Model with an interaction term (rs6500843:Chemotherapy)			
	HR	95% C.I.	p
rs6500843	0.93	0.84 - 1.04	0.210
Chemotherapy	0.60	0.40 - 0.92	0.018
Age	1.04	1.04 - 1.05	< 10^−16^
Grade	1.40	1.27 - 1.54	6.4 × 10^−12^
ER	0.74	0.64 - 0.86	7.3 × 10^−5^
T	1.53	1.39 - 1.69	< 10^−16^
N	2.03	1.78 - 2.32	< 10^−16^
rs6500843:Chemotherapy	1.33	1.12 - 1.58	9.6 × 10^−4^
*(Likelihood ratio test=651 on 11 df, p=0 n= 9680, number of events= 1176)*			

### eQTL analysis of SNPs in the survival-associated loci

To determine if rs6500483 or rs11155012, or other SNPs in the surrounding LD regions (r^2^ > 0.1), associate with gene expression in breast cancer, two publicly available data sets (containing both genotype and gene expression data) were used for expression quantitative loci (eQTL) analysis: TCGA [12], and METABRIC [13]. We classified eQTLs as *cis* if they occurred within ±1 Mb from any SNP in the LD region; any eQTLs outside these regions were classified as *trans*.

The SNP rs6500843 is located in chromosome 16, in an intron of the *RBFOX1* gene, which is the only gene in the immediate LD region surrounding the SNP. The expanded *cis*-eQTL region (±1 Mb) surrounding the SNP also contains the RNA coding genes *MIR8065*, *LINC01570*, and *LOC102723308*, as well as the uncharacterized gene *LOC440337*. No *cis*-eQTLs were observed in the rs6500843 locus. When we examined *trans*-eQTLs, the expression levels of six genes were associated with genotypes in the rs6500843 locus; *MCM3, CENPM, PKMYT1, AURKA, KIFC1, TK1* (Bonferroni-corrected for all genes on the array; p < 0.05). In an enrichment analysis for Gene Ontology biological processes, using an arbitrary raw eQTL p-value threshold of p < 10^−5^ to select an expanded candidate gene list, GO terms involved in cell cycle control, mitosis and DNA replication emerged as highly significant for the trans-eQTLs (34 probes representing 28 genes) in the rs6500843 locus (Table 3). Note that rs6500843 itself was not present in the data set, nor were any SNPs in complete LD with it. The vast majority of eQTLs in this region are associated with the SNPs rs6500842 (r^2^ 0.54, D' 0.87 with rs6500843) and rs7205424 (r^2^ 0.26, D' 0.53), suggesting a biologically significant role for the haplotype(s) tagged by these two SNPs.

**Table 3:** Pathway-enrichment analysis of putative trans-eQTLs associated with rs6500843. Only statistically significant GO terms are shown (BH-corrected p < 0.05) Genes with a statistically significant eQTL association are shown in bold; the remaining genes have been included using a more inclusive p-value threshold of p < 10^−5^. In total, 28 genes were included in the pathway enrichment analysis.

GO accession	description	N	Fold enr.	p	Genes
GO:0007049	cell cycle	15	10.90	1.88 × 10^−12^	EXO1, **KIFC1**, PRC1, BLM, FOXM1, **PKMYT1**, **AURKA**, BIRC5, CHEK2, UBE2C, **MCM3**, MCM6, UHRF1, KPNA2, CDCA5
GO:0022403	cell cycle phase	10	13.62	1.21 × 10^−8^	EX01, **KIFC1**, PRC1, BLM, **PKMYT1**, BIRC5, **AURKA**, UBE2C, KPNA2, COCAS
GO:0000279	M phase	9	15.42	4.01 × 10^−8^	EX01, **KIFC1**, PRC1, **PKMYT1**, BIRC5, **AURKA**, UBE2C, KPNA2, CDCA5
GO:0000278	mitotic cell cycle	9	13.71	9.94 × 10^−8^	**KIFC1**, PRC1, BLM, **PKMYT1**, BIRC5, **AURKA**, UBE2C, KPNA2, CDCA5
GO:0022402	cell cycle process	10	9.98	1.75 × 10^−7^	EX01, **KIFC1**, PRC1, BLM, **PKMYT1**, BIRC5, **AURKA**, UBE2C, KPNA2, CDCA5
GO:0006259	DNA metabolic process	8	8.91	1.43 × 10^−5^	EX01, UHRF1, BLM, POLO, **MCM3**, KPNA2, **TK1**, MCM6
GO:0007067	mitosis	6	15.37	2.88 × 10^−5^	**KIFC1**, **PKMYT1**, BIRC5, **AURKA**, UBE2C, CDCA5
GO:0000280	nuclear division	6	15.37	2.88 × 10^−5^	**KIFC1**, **PKMYT1**, BIRC5, **AURKA**, UBE2C, CDCA5
GO:0000087	M phase of mitotic cell cycle	6	15.10	3.14 × 10^−5^	**KIFC1**, **PKMYT1**, BIRC5, **AURKA**, UBE2C, CDCA5
GO:0048285	organelle fission	6	14.77	3.49 × 10^−5^	**KIFC1**, **PKMYT1**, BIRC5, **AURKA**, UBE2C, CDCA5
GO:0006260	DNA replication	5	14.83	2.71 × 10^−4^	BLM, POLO, **MCM3**, **TK1**, MCM6
GO:0051329	interphase of mitotic cell cycle	4	21.89	6.80 × 10^−4^	BLM, BIRC5, KPNA2, CDCAS
GO:0051325	interphase	4	21.27	7.39 × 10^−4^	BLM, BIRC5, KPNA2, COCAS
GO:0007017	microtubule-based process	5	11.14	7.99 × 10^−4^	**KIFC1**, PRC1, **AURKA**, UBE2C, KPNA2
GO:0051301	cell division	5	9.55	0.001414	**KIFC1**, PRC1, BIRC5, UBE2C, CDCA5
GO:0051726	regulation of cell cycle	5	8.51	0.002157	BLM, **PKMYT1**, BIRC5, CHEK2, UBE2C

The LD region around rs11155012 contains two genes: *CCDC28A* and *ECT2L*. In total, 34 genes are located in the expanded *cis-*eQTL region around this locus. One nominally significant *cis*-eQTL was observed in this region, and occurred as a positive correlation between the rare allele of rs9321678 (r^2^ = 0.147, D' = 1 with rs11155012) and the neighboring *CCDC28A* gene (*t* = 2.003, p = 0.0454). In the *trans*-eQTL analysis, the rs11155012 locus was associated with the expression of the genes *CHRFAM7A, CRYBB2, RASGRP4, MOCS1, SOX21,* and *UPK3B.* No statistically significant GO term enrichment was detected for the rs11155012 locus.

The complete *trans*-eQTL results for both loci are presented in detail in Supplementary Table 4.

### Expression based survival analysis of the genes associated with the rs6500483 and rs11155012 loci

Next, we utilized the Kaplan-Meier Plotter database [14] to investigate whether any of the genes surrounding our statistically significant SNPs are associated with breast cancer survival (10 year relapse-free survival) at the gene expression level. Regions of interest were defined as regions containing SNPs with r^2^> 0.2 to the nominal SNP. The region for rs11155012 contained two genes (*ECT2L, CCDC28A*), and the region for rs6500483 contained one (*RBFOX1/A2BP1*). These candidate genes were analyzed in the “any adjuvant chemotherapy” category, as information on specific types of chemotherapy was not available in the database. Among these cases (N = 425), high expression of the gene *CCDC28A* was associated with better prognosis (p = 0.00012, HR 0.5, 95% C.I. 0.35 - 0.72) (Supplementary Figure 1). It was also associated with prognosis in the full data set, irrespective of treatment (N = 3455, p = 0, HR 0.56, 95% C.I. 0.49 - 0.63). This is consistent with the results of the *cis*-eQTL analysis: the rare allele of rs9321678 was associated with increased CCDC28A expression, which in turn is associated with better prognosis. The rare alleles of rs11155012 and rs9321678 segregate in different haplotypes (negative correlation), as can be deduced from the LD information (r^2^ = 0.147, D' = 1, MAFs are roughly equal [0.20 and 0.18, respectively]). Consequently, the rare allele of rs11155012 would be expected to associate with worse prognosis, which is indeed the case in our SNP survival analysis. Of the trans-eQTL genes associated with the rs11155012 locus, the genes *CHRFAM7A, CRYBB2, MOCS1* and *SOX21* were associated with survival among all cases in the KM-Plotter analysis, whereas only one (*SOX21*) was associated with survival among chemotherapy-treated cases (Supplementary Table 5). Significant heterogeneity (p = 0.0044; 0.0239 after BH-adjustment for multiple testing) was observed for SOX21: high SOX21 associated with better prognosis among untreated cases (HR 0.82, 95% C.I. 0.69 – 0.98), but the effect was reversed among chemotherapy-treated cases (HR 1.45, 95% C.I. 1.02 – 2.06).

Expression of the *RBFOX1* gene at the rs6500483 locus did not associate with survival in the KM-plotter database, either among chemotherapy-treated cases or among all cases. In contrast, all six trans-eQTL genes associated with the rs6500843 locus (*MCM3, CENPM, PKMYT1, AURKA, KIFC1, TK1*) were associated with survival when analyzing all cases irrespective of treatment, as well as among cases who specifically had not received systemic chemotherapy. All other genes but *AURKA* associated also with survival among cases treated with adjuvant chemotherapy (Supplementary Table 5) which would suggest a treatment-independent effect for most of these genes. However, a test of heterogeneity between the treated and non-treated groups indicated a statistically significant difference in *AURKA*-associated survival between treated and non-treated cases (heterogeneity p=0.021 after BH-adjustment for multiple testing): while high *AURKA* expression associated with poor survival in untreated cases, no such effect was seen in chemotherapy-treated cases.

**Figure 3:**
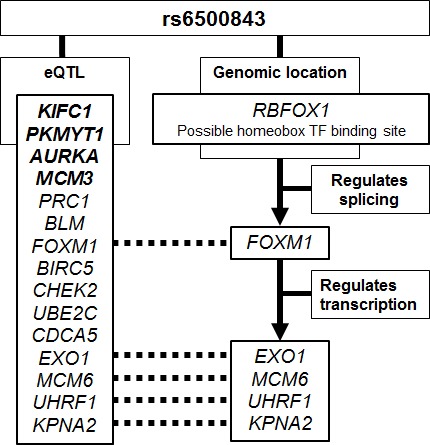
Schematic summary of the putative evidence connecting the rs6500843 SNP to the trans-eQTL SNPs associated with this locus The eQTL genes marked in bold are associated with the rs6500843 locus at a statistically significant level after conservative Bonferroni correction; the remaining genes listed here are associated with SNP genotype at p < 10-5, and also belong to the Gene Ontology group GO:0007049 (cell cycle) which was most strongly enriched in the DAVID analysis. RBFOX1 and FOXM1 target gene identification is based on previously published data [13, 14].

Next, we investigated the possible connection between the rs6500843 locus and the trans-eQTL genes. Rs6500843 is located in an intron of *RBFOX1*, a tissue-specific splicing regulator, for which target genes have been previously published [15]. Given that the genetic neighborhood around rs6500843 contains no other protein coding genes, and little is known about the three RNA coding genes in the region, we operated here under the hypothesis that the SNP is functionally associated with the *RBFOX1* gene. As splice variants of gene products may give rise to different signals in microarray experiments, we cross-referenced the published list of RBFOX1 targets against our expanded list of putative trans-eQTL genes (raw p < 10^−5^ in the eQTL analysis) that also fall within the enriched GO:0007049 (cell cycle) Gene Ontology group (Table 3), and came up with one gene: the transcription factor FOXM1. Genome-wide target genes for FOXM1 have also been previously published [16]; comparison of the statistically significant sites (combined genomic binding and coexpression) with our trans-eQTL list yielded four genes regulated by FOXM1: *EXO1, MCM6, UHRF1,* and *KPNA2*. These connections between the genomic locus and the eQTL genes have been schematically summarized in Figure 3.

### The rs11155012 locus contains a genomic regulatory region

Finally, we investigated the possibility that the prognostic SNPs identified in this study are in linkage disequilibrium with other, functionally significant variants in their genomic vicinity. All SNPs in linkage disequilibrium (r^2^> 0.2) with rs6500843 and rs11155012 were analyzed using HaploReg, a database of genomic regulatory elements [17]. Rs11155012 is in high LD with a number of SNPs affecting a genomic enhancer site (Supplementary Table 6) active in human mammary epithelial cells (HMEC). The site has been experimentally shown to bind the proteins FOXA1 and FOXA2, and a Foxa binding motif is predicted to be altered by rs6570291, a SNP in near-complete LD with rs11155012 (r^2^ = 0.99).

Equally strong evidence of an active regulatory site was not observed for the rs6500843 locus. The SNP itself is predicted to alter 33 transcription factor binding motifs, mostly homeobox-containing tissue-specific transcription factors, but no supporting experimental evidence is present in the HaploReg data.

## DISCUSSION

We have utilized three independent data sets to evaluate the significance of putative prognostic/predictive SNPs from an initial GWS pilot investigating survival after adjuvant chemotherapy in breast cancer. This analysis was carried out as a meta-analysis between iCOGS studies and two additional data sets (SUCCESS-A, POSH). Two loci emerged as statistically significant in the two-stage analysis: rs6500843 among cases treated with any chemotherapy, and rs11155012 among cases treated with anthracyclines. Of these, rs6500843 was found to interact with adjuvant chemotherapy in a subsequent multivariate interaction analysis, reinforcing the notion that its association with survival may have a predictive basis: carriers of the rs6500843 G-allele appear to have somewhat worse survival than other chemotherapy-treated cases.

The only gene in the LD region surrounding rs6500843 is *RBFOX1 (A2BP1),* which encodes a highly conserved RNA-binding protein involved in the control of tissue-specific mRNA splicing [18]. It has been identified as a common target of LOH and copy number variation in other types of malignancies [19-21], and a SNP in the gene (not in LD with rs6500843) has been reported to associate with survival in non-small-cell lung cancer [22], but little is known about its role in breast cancer. While the rs6500843 SNP is predicted to alter a homeobox transcription factor binding site targeted by 33 transcription factors, we did not find further experimentally verified evidence of a genetically modulated regulatory region in this locus, nor was *RBFOX1* gene expression itself associated with breast cancer survival. However, a *trans*-eQTL analysis of the SNPs in the rs6500843 region identified six statistically significant genes, all of which were associated with survival in breast cancer in the *Kaplan-Meier Plotter* analysis (and five in the chemotherapy-only subgroup). Notably, the Aurora-A kinase (*AURKA*) associated with differential survival when comparing chemoterapy-treated and non-treated cases. High *AURKA* expression appeared to associate with poor survival in untreated cases, whereas no such effect was seen in chemotherapy-treated cases, which suggests a favorable response to the chemotherapy regimens administered to these cases. *AURKA* has been previously suggested to be a master regulator of cellular radio- and chemoresistance, proliferation, cell cycle progression and anchorage-independent growth [23].

In order to detect enriched Gene Ontology biological processes among the eQTL genes, we relaxed the criteria of statistical significance and analyzed a slightly larger list of candidate genes (n = 28). This set of candidates was strongly enriched for genes involved in cell cycle control (GO: 0007049; *cell cycle*): 15 out of 28 genes fell within this category, an 11-fold enrichment compared to a random selection of genes. The set of genes includes *EXO1*, knockdown of which has been reported to desensitize H196 lung cancer cells to paclitaxel treatment [24]; *KIFC1*, reported to modulate docetaxel sensitivity [25]; the breast cancer susceptibility gene *CHEK2* [26, 27]; the Bloom syndrome gene *BLM* [28]; and the Survivin gene *BIRC5*, reported to associate with chemo- and radioresistance in breast cancer [29]. It is intriguing to see this group of cell cycle control and chemoresistance-associated genes emerge in eQTL with a SNP associated with survival after chemotherapy, especially as there is a plausible connection between the rs6500843 locus and this set of trans-eQTL genes (Figure 3). Rs6500843 is located in an intron of *RBFOX1*, a tissue-specific splicing regulator that targets the proto-oncogene *FOXM1*, a transcription factor involved in cell cycle control. In our analyses, *FOXM1* itself emerged as a putative trans-eQTL gene associated with the rs6500843 locus, and it has been reported to regulate four other genes on that list: *EXO1, MCM6, UHRF1,* and *KPNA2*. Of these, *EXO1* is of particular interest, as the FOXM1-EXO1 regulatory connection has been previously reported to modulate chemoresistance in ovarian carcinoma cells [30]. An important caveat to this analysis is that the SNP rs6500843 itself was not present in the eQTL data set, and the eQTL results are based on SNPs in incomplete linkage disequilibrium with it. The functional significance of rs6500843 itself, and the putative transcription factor binding site it resides in, therefore remains unclear.

The other SNP emerging as statistically significant in the validation analysis, rs11155012, is located in a LD region that contains two genes, *CCDC28A* and *ECT2L*, the biological functions of which are poorly understood. Our analysis of publicly available gene expression data [12] indicates that *CCDC28A* transcript abundance is associated with relapse-free survival in breast cancer, which suggests that rs11155012 may be linked to a prognostically significant functional variant in this gene. Indeed, rs11155012 is in LD with a number of SNPs affecting a genomic enhancer site active in human mammary epithelial cells. The site has been experimentally shown to bind the proteins FOXA1 and FOXA2, and a Foxa binding motif is predicted to be altered by rs6570291, a SNP in near-complete LD with rs11155012 (r^2^ = 0.99). FOXA1 (Hnf-3-alpha) is a key transcription factor involved in mechanisms known to be critical in breast carcinogenesis: estrogen receptor -mediated signaling, and cell cycle control in conjunction with BRCA1 [31, 32]. Furthermore, a modest *cis*-eQTL effect was observed between rs9321678 (r^2^ = 0.147, D' = 1 with rs11155012) and the *CCDC28A* gene. We also detected a trans-eQTL between rs9495127 (r^2^ = 0.129, D' = 1) and the transcription factor *SOX21*, a gene whose expression associates with increasing hazard among chemotherapy-treated cases despite being protective in untreated cases. While causality cannot be established without experimental work, these findings provide supporting evidence for an association between genetic variation in this regulatory site and breast cancer survival. The predictive value of this genetic locus remains questionable, however, as we did not observe an interaction between rs11155012 and treatment in the clinical data.

The hazard ratios for rs6500483 show some heterogeneity between the data sets used in this study: SUCCESS-A appears to disagree with the other data sets. Rs6500483 is represented in SUCCESS-A by rs4786939, but these two SNPs are in nearly complete linkage disequilibrium (r^2^ 0.967, D' 1.0), indicating that the use of a tagging SNP is an unlikely source of heterogeneity here. Heterogeneity between follow-up times and treatment regimens would be a more likely reason: the cases in SUCCESS-A have all received taxane-based therapy, a regimen poorly represented in the other data sets. Unfortunately, the specific chemotherapy agents involved in the genetic association cannot be resolved using our currently available data. Additionally, in light of our use of GWAS as a starting point, it must be noted that our findings do not reach genome-wide significance. However, the purpose of the initial Stage I GWAS was to define a candidate SNP set that would most likely capture any true survival-associated SNPs, the statistical significance of which would then be evaluated in the substantially more powerful Stage II analysis. We do not anticipate very large effect sizes in SNP-based survival analyses, and therefore utilized a stepwise study design to alleviate the problem of multiple testing and resultant loss of statistical power. Only 39 SNPs were analyzed in Stage II, two of which meet our criteria of statistical significance. Both findings are supported by additional lines of evidence from gene expression- and regulome-based analyses. Nevertheless, these results must be viewed as exploratory and hypothesis-generating in nature.

In conclusion, we have conducted a two-stage genetic association study to detect SNPs associated with survival after chemotherapy. One SNP, rs6500843, was found to interact specifically with adjuvant chemotherapy, independently of standard prognostic markers, whereas rs11155012 may associate with survival in general. eQTL analysis of the rs6500843 locus identified a group of genes known to associate with cancer progression, chemoresistance, and survival. We propose that these genes may be connected to the rs6500843 locus via a RBFOX1-FOXM1 -mediated regulatory pathway. If confirmed, these findings may aid in the development of better predictive markers and improved individualized cancer therapy.

## MATERIALS AND METHODS

### Study design

Initially, a genome-wide study (Stage I: HEBCS-GWS) was conducted to discover candidate SNPs that may be associated with breast cancer survival, with an emphasis on treatment-based subgroups. This candidate SNP set was then analyzed in three validation datasets (Stage II: iCOGS, SUCCESS-A, POSH). SNPs emerging as statistically significant within Stage II were further examined in an eQTL analysis of two breast cancer data sets (TCGA, METABRIC), supported by *in silico* genomic feature analysis. The data sets used in this study are described below, and summarized in Table 4. Informed consent has been obtained from all patients included in this study.

**Table 4:** Description of the data sets used in this study

	HEBCS GWS	POSH GWS	SUCCESS-A	iCOGS
**No. of cases**	805	536	3596	17828
**Vital status**				
Alive	466 (58%)	300 (56%)	3389 (94%)	15630 (88%)
Deceased: all-cause	339 (42%)	236 (44%)	207 (6%)	2198 (12%)
**Follow-up mean ±SD (years)**	10.6 ± 6.6	4.1 ± 2.0	3.9 ± 1.7	7.3 ± 4.0
**Age at diagnosis, mean [range)**	54.1 [22 - 87]	35.8 [18 - 41]	53.6 [19 - 85]	55.2 [19 — 95]
**ER**				
Negative	230 (29%)	370 (69%)	1106 (31%)	3002 (17%)
Positive	513 (64%)	165 (31%)	2458 (68%)	11753(66%)
Missing data	62 (8%)	1 (0.2%)	32 (1%)	3073 (17%)
**Grade**				
1	144 (18%)	13 (2%)	165 (5%)	2911 (16%)
2	312 (39%)	84 (16%)	1698 (47%)	6354 (36%)
3	280 (35%)	422 (79%)	1698(47%)	4414 (25%)
Missing data	69 (9%)	17 (3%)	35 (1%)	4149 (23%)
**T / tumor size category ^a^**				
1	390 (48%)	232 (43%)	1464(41%)	9338 (52%)
2	304 (38%)	236 (44%)	1856 (52%)	4615 (26%)
3	50 (6%)	49 (9%)	192 (5%)	635 (4%)
4	47 (6%)	12 (2%)	50 (1%)	*n/a*
Missing data	14 (2%)	7(1%)	34 (1%)	3240 (18%)
**N (nodal metastasis)**				
Negative	338 (42%)	248 (46%)	1248 (35%)	8976 (50%)
Positive	446 (55%)	262 (49%)	2311 (64%)	5471 (31%)
Missing data	21 (3%)	26 (5%)	37 (1%)	3381 (19%)
**M (distant metastasis)**				
Negative	740 (92%)	481 (90%)	3487 (97%)	2834 (16%)
Positive	57 (7%)	50 (9%)	4 (0.1%)	267 (1.5%)
Missing data	8 (1%)	5(1%)	105 (2.9%)	14727(83%)
**Adjuvant chemotherapy treatment**				
No adjuvant chemotherapy	445 (55%)	18 (3.4%)	0 (0%)	11108 (62%)
Anthracycline+Taxane	14 (2%)	129 (24%)	3596 (100%)	733 (4%)
Anthracycline	191 (24%)	375 (70%)	-	2277 (13%)
Taxane	2 (0.2%)	8 (1.5%)	-	135 (0.8%)
Methotrexate	153 (19%)	4 (0.7%)	-	1022 (6%)
Missing data	-	-	-	2528 (14%)
**Adjuvant Endocrine treatment**				
Treated	282 (35%)	183 (34%)	2458 (68%)	11340 (64%)
Not treated	520 (65%)	344 (64%)	1138 (32%)	5670 (32%)
Missing data	3 (0.4%)	9 (1.7%)	-	818 (5%)

a T was acquired from TNM staging for HEBCS, POSH, SUCCESS-A, and derived from tumor diameter in COGS (1: s2crn. 2: >2cm and s5cm, 3: >5cm); T4 (inflammatory carcinoma) is therefore undefined for COGS.

### Discovery GWS (Stage I: HEBCS-GWS)

Genotype information was obtained from a study series consisting of 805 Finnish breast cancer cases (HEBCS-GWS). All cases were female, ascertained for their first primary invasive breast cancer. Of these, 423 cases originated from a prospective patient series of consecutive unselected, incident breast cancer patients treated in the Helsinki University Hospital Department of Oncology in years 1997–1998 and 2000 [33, 34]. The series also includes 140 cases recruited between 2001 and 2004, and 242 additional familial cases [7, 35]. To increase the statistical power of survival analyses, the GWS series was specifically enriched for cases with reduced survival (distant metastasis or death at the time of the initiation of the study in 2008): in total, the series includes 312 breast cancer specific events, and 339 any-cause mortality events at the time of analysis. The cancer diagnoses were confirmed through the Finnish Cancer Registry and hospital records. Information on the date and cause of death was obtained from the Finnish Cancer Registry, a central database of diagnostic and death information on all cancer patients in Finland.

The HEBCS-GWS series (“Stage I”) was genotyped using the Illumina 550K platform as previously described [36]. The preliminary survival analysis was carried out using three different endpoints in parallel: five-year BDDM (breast cancer death or distant metastasis), 10-year breast cancer specific survival, and 10-year overall survival, using a codominant genetic model (test for heterogeneity between genotypes). Since survival after chemotherapy was the main focus of this study, it would have been ideal to entirely restrict the initial Stage I analysis only to chemotherapy-treated cases (comprising 45% of the data set; Table 4), given sufficient statistical power. To determine the feasibility of this approach, the R package *‘survSNP’* was employed to estimate the statistical power of our survival analysis [37]. Using a significance threshold of p < 10^−5^ and a hypothetical SNP with a minor allele frequency of 0.25 as the *ad hoc* target for discovery, we had 80% power to detect a per-allele HR of 1.6 in the whole sample set, but only 32% power if the analysis was restricted to chemotherapy-treated cases only. Analysis of specific chemotherapy regimens would have decreased the statistical power further. Based on this, we concluded that a treatment-based subgroup analysis alone would be unacceptably underpowered. Therefore, to be eligible for subsequent validation, SNPs had to meet one of these three more inclusive criteria: 1) main effect p < 10^−4^ and p < 0.01 among chemotherapy-treated cases, 2) p < 10^−4^ among chemotherapy-treated cases, or 3) p < 10^−3^ among chemotherapy-treated cases and a homozygote-associated hazard ratio > 3.0. These SNPs (n = 45) were then selected as candidates for Stage II validation and genotyping as part of the iCOGS project [2].

### Validation studies (Stage II: iCOGS, POSH, SUCCESS-A)

The candidate SNPs from Stage I were analyzed in three additional data sets: iCOGS, POSH, SUCCESS-A (described in detail below).

### iCOGS

SNPs identified as putatively significant in Stage I HEBCS GWAS analysis of chemotherapy-treated cases were included on a custom Illumina Infinium array (iCOGS) for large scale genotyping of a data set of 50927 individuals from 52 Breast Cancer Association Consortium (BCAC) member studies, 41 of which were of predominantly European ancestry, 9 of Asian, and 2 of African-American ancestry. Genotyping and quality control was carried out as previously described [2].

Studies represented on the iCOGS chip were included in survival analysis here if sufficient follow-up data was available, with a minimum requirement of at least 10 survival events (deaths from any cause) per study. Additionally, we included only studies with cases from predominantly European ancestry, and from which adjuvant chemotherapy information was available. At the individual level, we included only female cases ascertained for their first primary, invasive tumor. After these filtering steps, the data set consisted of 17828 eligible cases (2198 any-cause deaths; 12%), of which 6720 cases were treated with any adjuvant chemotherapy. Of these, 3010 cases were known to have been treated with anthracycline-based chemotherapy. While data on taxane (n = 868) and methotrexate-based (n = 1022) adjuvant treatment was also available, these treatments were not analyzed as separate subgroups due to the small sample sizes and poor comparability with the other data sets (Table 4). For 2528 cases, the specific type of chemotherapy regimen was unknown. The cases included here come from 16 separate BCAC studies, which have been described in Supplementary Table 7.

### POSH GWS

The POSH GWS consisted of 536 individuals (236 any-cause deaths; 44%) selected from a consecutively ascertained cohort of early onset cases (diagnosed with invasive breast cancer at the age of 40 or earlier) from the United Kingdom. These cases were enrolled into the *Prospective study of Outcomes in Sporadic versus Hereditary breast cancer* (POSH) between 2000 and 2008, as previously described [38]. The POSH GWS material was enriched for cases with poor prognosis by including cases with triple-negative breast cancer (n=401), and cases with particularly short duration of breast cancer survival (<2 years, n=48); additional cases with relatively long survival were then included for contrast (>4 years, n=125) [39]. These cases were genotyped on the Illumina 660-Quad SNP array; genotyping and quality control was carried out as previously described [39]. 518 cases (97%) in this data set were treated with adjuvant chemotherapy.

### SUCCESS-A

SUCCESS-A is a randomized Phase III study of response to treatment of early primary breast cancer with adjuvant therapy after surgical resection. The cases are all treated with anthracycline- and taxane-based adjuvant chemotherapy, and randomized for Gemcitabine treatment. The sample set consisted of 3596 cases (207 any-cause deaths; 6%) that were recruited between 2005 and 2007 from 250 study sites across Germany. Genotyping was carried out using the Illumina Human OmniExpress-FFPE BeadChip platform. The SUCCESS-A data as well as detailed descriptions of the materials and methodology can be acquired from the NCBI database of Genotypes and Phenotypes (dbGaP Study Accession: phs000547.v1.p) [40].

### Statistical analysis

In stage II, unless otherwise noted, 10-year overall survival (death from any cause) was used as the end point in all survival analyses, as all-cause mortality data was the most widely available in the iCOGS data set. This also allowed us to avoid the potential biases that can arise from clinical follow-up data in multicenter studies [41].

All survival analyses were run under the additive genetic model, follow-up times left-truncated, and adjusted for patient age at diagnosis, and (in the case of iCOGS) stratified by study in order to minimize potential biases arising from differing case recruitment strategies and different populations of origin. The analyses were performed within the same subgroups (chemotherapy-treated cases and anthracycline-specific analysis) as in stage I.

To alleviate potential biases arising from population stratification, e.g. cryptic relatedness of cases, the analysis was also adjusted for three genetic principal components to correct for possible population substructure. The principal components were calculated from the genotypes of uncorrelated (r^2^ < 0.2) SNPs in the genome-wide data sets. Briefly, for the HEBCS-GWS, POSH and SUCCESS-A data sets, we pruned a subset of SNPs not in substantial LD with any other SNPs (r^2^< 0.2) and ran a principal component analysis (PCA) using the ‘*GenABEL’* package in R. The PCA analysis for iCOGS has been described previously [2]; as with the other three data sets, only three principal components were used in this study.

The selected SNPs from the HEBCS-GWS data set (Stage I) were also re-analyzed at this point using the same parameters as with the validation data. This means that Stage I results were here re-analyzed only under the additive model (which can be expected to capture both dominant and recessive signals), regardless of the original selection criteria, reducing the amount of multiple testing in the final meta-analysis stage. While the original SNP selection was based on more inclusive criteria, the purpose of this was to ensure that the final results are directly comparable across the stages of the study. Thus, allele calling and quality control for HEBCS-GWS was performed as previously described [39], and the survival analysis was performed as described above. Similar to the validation data sets, principal component analysis (PCA) was included for HEBCS-GWS as well at this stage.

Results of the Stage II (iCOGS, POSH, SUCCESS-A) survival analysis were meta-analyzed under a fixed-effects model using the *‘rmeta’* package in R, and the resulting meta-statistics were used to determine statistical significance. Benjamini-Hochberg correction was applied to account for multiple testing [42].

Statistically significant SNPs in treatment-based subgroups were further tested for interaction with treatment in the iCOGS data set, as it contains both treated and non-treated cases in abundant numbers, enabling robust interaction testing. This analysis was adjusted for clinically relevant covariates (grade, ER, tumor size category [T], nodal status [N], age at diagnosis). Distant metastasis at diagnosis (M) was not included as a covariate, as this information was missing for the majority of cases. To avoid biases introduced by mostly missing data, cases known to be M1 were not specifically excluded from the analysis either. Two Cox proportional hazards models were generated, one with the SNP and treatment status as independent covariates, and one that also included an interaction term between genotype and treatment. The statistical significance of an interaction was then determined using a likelihood-ratio test between the two models. To rule out confounding effects caused by other treatment types, we also conducted a secondary interaction analysis where radiotherapy (yes/no), surgery (yes/no), and adjuvant endocrine therapy (yes/no) were added as additional covariates in the model.

We employed chi-square tests of heterogeneity to test for association between SNP genotypes and the following clinicopathological characteristics: estrogen receptor status (ER; positive or negative), histological grade (1 – 3), tumor size (T; 1 – 4), and lymph node metastasis (N; positive or negative) in the iCOGS data set.

### eQTL analysis

To determine if the survival-associated SNPs or other SNPs in the LD region (r^2^ > 0.1) associate with gene expression in breast cancer, two publicly available data sets (containing both genotype and gene expression data) were used for eQTL analysis: TCGA [12], and METABRIC [13]. The TCGA gene expression data is mRNA sequencing data from the Illumina HiSeq 2000 platform, whereas the METABRIC gene expression data was generated by the Illumina Human WG6 v3 platform. Tumor tissue genotyping had been carried out using the Affymetrix Genome Wide Human SNP array 6.0 for both data sets. Of the two validated SNPs, rs11155012 itself was represented on this SNP array, whereas rs6500843 was not. The latter was best represented by the linked SNPs rs4786121 and rs7190693 (r^2^ 0.603, D' 1.0 for both SNPs); note that all SNPs in the LD region with r^2^ > 0.2 were nevertheless included in the eQTL analysis. The TCGA breast cancer data set consists of 913 solid breast tumor samples that also had genotype data available. METABRIC consists of 1328 solid breast tumor samples with both genotype and gene expression data. eQTL analysis was carried out by calculating linear models between genotype and gene expression using the R package *‘MatrixEQTL’* [43]. Only genes present in both data sets were included in the analysis: the TCGA and METABRIC data sets were analyzed separately and then meta-analyzed to detect the most consistent results. We searched for *cis*-eQTLs within regions defined as ±1 Mb from any SNP in the LD region; no formal multiple testing correction was applied to eQTL results within these regions. Any genes outside these regions were analyzed for *trans*-eQTL and subjected to transcriptome-wide multiple testing correction (Bonferroni).

In addition to looking for individual genes that are statistically significant in the eQTL analysis, we also ran a pathway-enrichment analysis on a set of candidate genes selected using a more lenient p-value threshold from the eQTL analysis (raw p < 10^−5^). The analysis was restricted to Gene Ontology Biological Processes and was performed using the DAVID Functional Annotation Tool [44, 45].

### Further *in silico* evaluation of SNPs and candidate genes

It is likely that the prognostic SNPs identified in this study are merely linkage disequilibrium proxies for other, functionally significant variants in their genomic vicinity. In an effort to identify the SNPs and genes with a direct functional impact on breast cancer survival, we utilized a number of public databases. For these analyses, regions of interest were defined as regions containing SNPs in linkage disequilibrium with the survival-associated SNPs at r^2^> 0.1. Candidate genes in the regions surrounding the prognostic SNPs, as well as statistically significant trans-eQTL genes associated with the survival-associated loci, were analyzed in the Kaplan-Meier plotter (http://kmplot.com/analysis/), a gene expression and survival database currently consisting of 4142 breast cancer cases, of which 425 were known to have been treated with adjuvant chemotherapy [12]. These analyses were performed using 10-year relapse-free survival, as this was the only end point available for the majority of cases in the Kaplan-Meier plotter data set, and optimized break points for the binarization of gene expression levels. All SNPs in these regions were further analyzed for their impact on regulatory features using HaploReg, a database of genomic functional annotations [15].

## SUPPLEMENTARY MATERIAL, TABLES AND FIGURE


