# Supplementary material for: The SNP rs6500843 in 16p13.3 is associated with survival specifically among chemotherapy-treated breast cancer patients
Source: Oncotarget. 2015 Mar 10;6(10):7390–407. doi: 10.18632/oncotarget.3506 (PMC4480688; doi:10.18632/oncotarget.3506)
Supplement: Supplementary file 1 [file oncotarget-06-7390-s001.pdf]

The SNP rs6500843 in 16p13.3 is associated with survival specifically among chemotherapy-treated breast cancer patients

Supplementary Material

a)

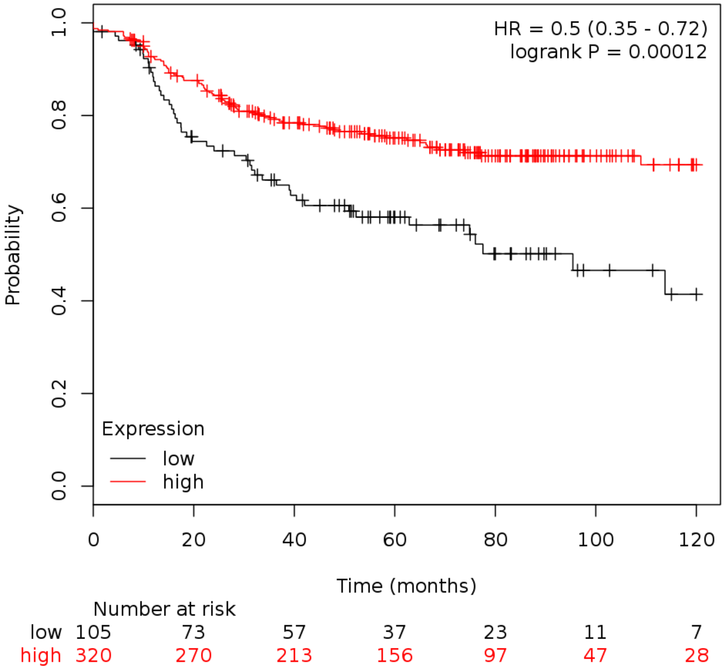

b)

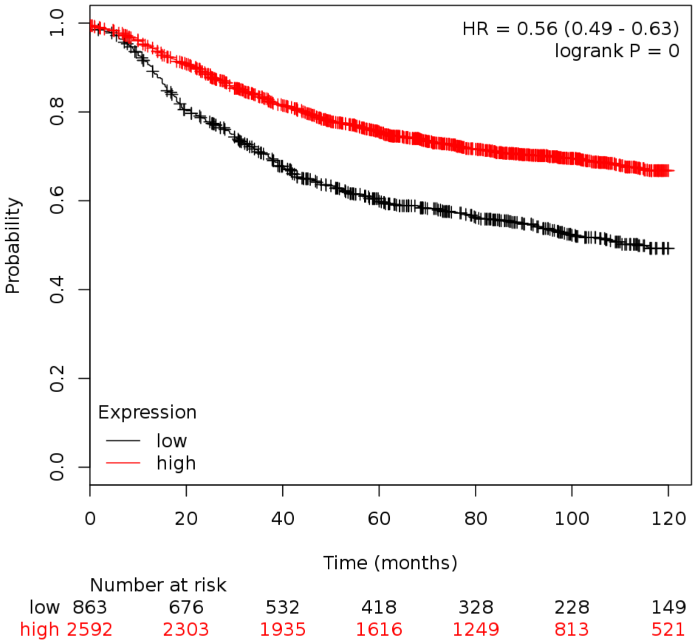

Supplementary Figure 1: Expression of the *CCDC28A* gene is associated with relapse-free survival in breast cancer. Kaplan-Meier curves according to binarized *CCDC28A* gene expression in the KM-plotter database: **a**) cases treated with adjuvant chemotherapy, **b**) all cases.

Supplementary Table 1: Summarized SNP selection and survival analysis statistics from Stage I and Stage II analyses for all SNPs. Note that some Stage I SNPs are represented by several LD-tagged SNPs in Stage II and vice versa, and as a result appear as duplicates in the table. "iCOGS_SNP", "POSH_SNP" and "SUCCESS_SNP" indicate the SNPs matching to the selected SNP from HEBCS-GWS. In cases where the SNP is a tagging SNP, LD information is provided (r2 > 0.7 and D' > 0.8). "NA" indicates that no satisfactory match was found in that data set. The "Cat." column indicates one of the following Stage I selection criteria: Ia) 10 year overall survival (p < 10-4); Ib) 10 year breast cancer specific survival (p < 10-4); Ic) 5 year BDDM (breast cancer death or distant metastasis, p < 10-4); II) chemotherapy-treated cases (p < 10-4); III) chemotherapy-treated cases with homozygote-associated HR > 3.0 (p < 10-3).

HEBCS_SNP	Cat.	S1_p_all	S1_p_chemo	R2	D'	iCOGS_SNP	POSH_SNP	SUCCESS_SNP
rs10004399	I	3.42E-07	1.42E-06	1	1	rs10004399	rs10004399	NA
rs10118669	Ib	3.99E-05	2.43E-02	1	1	rs10118669	rs10118669	rs7854237
rs11130876	Ib	5.59E-05	2.58E-03	1	1	rs11130876	rs11130876	NA
rs1603867	Ib	2.49E-05	4.10E-03	0.775	0.926			
rs6769168	Ic	2.03E-05	1.33E-03	0.739	0.889			
rs11155012	III	1.21E-04	6.80E-04	1	1	rs11155012	rs11155012	rs11155012
rs2182424	Ib	1.59E-06	2.34E-03	1	1	rs12569077	rs2182424	rs2182424
rs12693085	II	4.13E-02	5.13E-06	1	1	rs12693085	rs12693085	rs12693085
rs13019468	II	2.62E-03	2.33E-06	1	1	rs13019468	rs13019468	rs13019468
rs10164900	II	ns	5.29E-05	0.732	0.897			
rs11679968	II	ns	9.70E-06	0.732	0.897			
rs13063401	Ia	1.57E-07	1.44E-03	1	1	rs13063401	rs13063401	rs13063401
rs6791223	Ia	1.44E-07	1.76E-03	0.93	1			
rs10511205	Ia	3.13E-07	7.10E-03	0.866	1			
rs13323146	Ib	4.17E-05	2.37E-04	1	1	rs13323146	rs13323146	rs13323146
rs10511205	Ia	3.13E-07	7.10E-03	1	1	rs1515534	rs10511205	rs10511205
rs6791223	Ia	1.44E-07	1.76E-03	0.931	1			
rs13063401	Ia	1.57E-07	1.44E-03	0.866	1			
rs1492872	Ia	6.07E-06	2.06E-04	1	1	rs1602757	rs1492872	rs1492872
rs1603867	Ib	2.49E-05	4.10E-03	1	1	rs1603867	rs1603867	rs4686025
rs11130876	Ib	5.59E-05	2.58E-03	0.775	0.926			
rs1359280	Ia	4.55E-05	2.31E-03	1	1	rs1657224	rs1359280	rs7071602
rs2477017	Ia	4.74E-05	1.98E-03	0.962	1			
rs2490806	Ia	4.77E-05	1.98E-03	0.962	1			
rs16959669	II	1.05E-02	1.55E-05	1	1	rs16959669	rs16959669	rs16959669
rs1435737	II	1.26E-02	1.55E-05	1	1			
rs7179135	II	1.03E-02	1.55E-05	1	1			
rs17199739	III	3.12E-02	1.37E-04	0.832	1	rs17794824	rs996418	rs17199690
rs886108	III	3.12E-02	1.37E-04	0.832	1			
rs182286	Ib	3.79E-05	1.36E-03	1	1	rs182286	rs182286	rs182286
rs6769168	Ic	2.03E-05	1.33E-03	0.806	0.961			
rs1552329	Ia	1.10E-05	8.45E-03	1	1	rs1893791	rs1552329	rs1893791
rs2490806	Ia	4.77E-05	1.98E-03	0.781	1	rs2476995	rs2490806	rs2490817
rs2477017	Ia	4.74E-05	1.98E-03	0.781	1			

rs1359280	Ia	4.55E-05	2.31E-03	0.752	1			
rs2670927	III	2.22E-02	8.66E-04	1	1	rs2670927	NA	rs2670927
rs4891218	Ia	3.67E-07	7.46E-03	0.847	0.945	rs2959164	rs4891218	rs2959167
rs4307960	III	8.47E-05	1.13E-04	1	1	rs4307960	rs4307960	rs4307960
rs4502225	Ia	3.97E-05	3.75E-03	1	1	rs4502225	rs4502225	rs4502225
rs4656879	Ia	1.67E-07	1.27E-03	1	1	rs4656879	rs4656879	rs4656879
rs4712523	Ia	4.83E-05	5.06E-03	1	1	rs4712523	rs4712523	rs4712523
rs4891218	Ia	3.67E-07	7.46E-03	1	1	rs4891218	rs4891218	NA
rs9319675	Ia	6.10E-07	7.71E-03	0.8	1			
rs487989	III	ns	1.97E-04	0.866	1	rs526502	rs487989	NA
rs6077999	II	ns	8.48E-05	1	1	rs6077999	NA	rs6077999
rs487989	III	ns	1.97E-04	0.929	1	rs626802	rs487989	rs1858783
rs11865240	Ic	6.98E-05	8.60E-03	0.72	0.923	rs6500843	rs6500843	rs4786939
rs6750002	Ia	1.72E-05	3.29E-03	1	1	rs6750002	rs6750002	rs673811
rs12474326	Ia	8.64E-05	6.67E-03	0.962	1			
rs7080287	Ic	7.48E-05	3.28E-04	1	1	rs7080287	NA	rs7080287
rs7821832	III	3.84E-04	2.08E-04	1	1	rs7821832	rs7821832	rs7821832
rs6997996	III	3.78E-04	1.62E-04	1	1			
rs9319675	Ia	6.10E-07	7.71E-03	1	1	rs9319675	rs9319675	rs4891220
rs4891218	Ia	3.67E-07	7.46E-03	0.8	1			
rs964519	II	ns	4.24E-08	1	1	rs964519	rs964519	rs964519
rs6769168	Ic	2.03E-05	1.33E-03	0.967	1	rs9850025	rs6769168	rs9850025
rs182286	Ib	3.79E-05	1.36E-03	0.843	1			
rs11130876	Ib	5.59E-05	2.58E-03	0.771	0.893			
rs9857979	III	ns	4.65E-04	1	1	rs9857979	rs9857979	NA
rs1488688	Ia	6.34E-05	6.62E-03	1	1	rs9889588	rs1488688	rs3829957
rs1488688	Ia	6.34E-05	6.62E-03	0.877	1	rs9910285	rs1488688	NA
rs9932176	Ia	8.12E-09	7.22E-03	1	1	rs9932176	rs9932176	rs9932176

Supplementary Table 2: Tumor histopathological characteristics tabulated according to rs6500843 and rs11155012 genotype.

a) rs6500843

		GG	(%)	AG	(%)	AA	(%)	p-value
**ER**								
	negative	1121	(20.1%)	1423	(20.4%)	458	(20.8%)	0.7874
	positive	4453	(79.9%)	5555	(79.6%)	1744	(79.2%)	
**PgR**								
	negative	1535	(31.6%)	1879	(30.7%)	569	(29.3%)	0.1761
	positive	3323	(68.4%)	4250	(69.3%)	1371	(70.7%)	
**N (node status)**								
	negative	3378	(62.0%)	4251	(62.5%)	1346	(61.2%)	0.5354
	positive	2069	(38.0%)	2549	(37.5%)	853	(38.8%)	
**Grade**								
	1	1059	(20.5%)	1414	(21.8%)	438	(21.6%)	0.2988
	2	2413	(46.7%)	3022	(46.6%)	919	(45.3%)	
	3	1692	(32.8%)	2049	(31.6%)	673	(33.2%)	
**T (tumor size category)[a]**								
	1	3499	(63.3%)	4419	(64.1%)	1419	(65.4%)	0.2325
	2	1791	(32.4%)	2156	(31.3%)	668	(30.8%)	
	3	236	(4.3%)	317	(4.6%)	82	(3.8%)	
**HER2**								
	negative	2588	(84.8%)	3290	(85.2%)	1061	(86.6%)	0.3056
	positive	465	(15.2%)	570	(14.8%)	164	(13.4%)	

b) rs11155012

		GG	(%)	AG	(%)	AA	(%)	p-value
**ER**								
	negative	1649	(20.8%)	1142	(19.9%)	203	(19.4%)	0.3486
	positive	6285	(79.2%)	4587	(80.1%)	843	(80.6%)	
**PgR**								
	negative	2177	(31.3%)	1523	(30.5%)	268	(28.9%)	0.2867
	positive	4787	(68.7%)	3469	(69.5%)	660	(71.1%)	
**N (node status)**								
	negative	4820	(62.1%)	3513	(62.3%)	611	(61.0%)	0.7177
	positive	2942	(37.9%)	2123	(37.7%)	391	(39.0%)	
**Grade**								
	1	1558	(21.2%)	1114	(21.0%)	225	(23.0%)	0.4047
	2	3403	(46.2%)	2473	(46.7%)	464	(47.3%)	
	3	2397	(32.6%)	1711	(32.3%)	291	(29.7%)	
**T (tumor size category)[a]**								
	1	5011	(63.8%)	3675	(64.6%)	620	(61.9%)	0.2855
	2	2483	(31.6%)	1787	(31.4%)	332	(33.1%)	
	3	357	(4.5%)	228	(4.0%)	50	(5.0%)	
**HER2**								
	negative	3687	(85.1%)	2704	(85.5%)	519	(84.8%)	0.8655
	positive	644	(14.9%)	459	(14.5%)	93	(15.2%)	

Supplementary Table 3: Multivariate Cox proportional hazards model constructed to detect interaction between the SNP rs11155012 and treatment (anthracycline-based adjuvant chemotherapy).

Model without interaction

	HR	95% C.I.			p
rs11155012	1.162	1.05	-	1.29	3.90E-03
Anthracycline	0.993	0.81	-	1.22	9.50E-01
Age	1.05	1.04	-	1.06	0.00E+00
Grade	1.375	1.24	-	1.53	5.50E-09
ER	0.726	0.61	-	0.86	2.70E-04
T	1.535	1.37	-	1.72	9.70E-14
N	1.888	1.64	-	2.18	0.00E+00

(Likelihood ratio test=526 on 10 df, p=0, n= 8495, number of events= 907)

Model with an interaction term (rs11155012:Anthracycline)

	HR	95% C.I.			p
rs11155012	1.134	1.01	-	1.27	3.00E-02
Anthracycline	0.806	0.51	-	1.28	3.60E-01
Age	1.05	1.04	-	1.06	0.00E+00
Grade	1.374	1.23	-	1.53	5.70E-09
ER	0.728	0.61	-	0.86	3.00E-04
T	1.537	1.37	-	1.72	8.30E-14
N	1.887	1.63	-	2.18	0.00E+00
rs11155012:Anthracycline	1.141	0.88	-	1.48	3.20E-01

(Likelihood ratio test=526 on 11 df, p=0, n= 8495, number of events= 907)

Likelihood ratio test between models: chisq=0.98, df=1, p = 0.3222

Supplementary Table 4: Complete eQTL analysis results for all SNPs in the a) rs6500843 and b) rs11155012 locus. SNPs with r2 > 0.1 were included in the analysis.

a) SNPs in the rs6500843 region

SNP	Chr	Pos	rsq	D'	probe	gene	g_chr	gene_pos	p_value	
rs6500842	16	6808287	0.541	0.87	ILMN_1806818	MCM3	6	52237346	5.94E-08	*
rs7205424	16	6805109	0.255	0.531	ILMN_2368718	CENPM	22	40664777	1.36E-07	*
rs7205424	16	6805109	0.255	0.531	ILMN_1668814	CENPM	22	40669561	5.13E-07	*
rs7205424	16	6805109	0.255	0.531	ILMN_1766658	PKMYT1	16	2964552	1.25E-06	*
rs7205424	16	6805109	0.255	0.531	ILMN_1680955	AURKA	20	54378046	1.37E-06	*
rs7205424	16	6805109	0.255	0.531	ILMN_2222008	KIFC1	6	33482588	1.59E-06	*
rs7205424	16	6805109	0.255	0.531	ILMN_1806037	TK1	0	73682076	1.70E-06	*
rs7500396	16	6843128	0.104	0.358	ILMN_1787237	RELT	11	72765380	2.30E-06	*
rs11648981	16	6804820	0.255	0.531	ILMN_1709484	BLM	15	89155580	2.93E-06	
rs7205424	16	6805109	0.255	0.531	ILMN_2265654	UBE2C	20	43877439	3.14E-06	
rs7205424	16	6805109	0.255	0.531	ILMN_2357438	AURKA	20	54378054	3.28E-06	
rs7205424	16	6805109	0.255	0.531	ILMN_2368721	CENPM	22	40672375	3.37E-06	
rs7205424	16	6805109	0.255	0.531	ILMN_2401436	PKMYT1	16	2964004	3.66E-06	
rs11648981	16	6804820	0.255	0.531	ILMN_1673721	EXO1	1	240119417	3.80E-06	
rs7205424	16	6805109	0.255	0.531	ILMN_1728934	PRC1	15	89310505	3.86E-06	
rs12446260	16	6748770	0.101	0.504	ILMN_1681802	GRK6	5	176800596	4.05E-06	
rs7205424	16	6805109	0.255	0.531	ILMN_1782743	LETMD1	12	49739813	4.14E-06	
rs6500842	16	6808287	0.541	0.87	ILMN_2344971	FOXM1	12	2837420	4.19E-06	
rs6500842	16	6808287	0.541	0.87	ILMN_1678300	KPNA2	17	1716044	4.35E-06	
rs7205424	16	6805109	0.255	0.531	ILMN_1740291	POLQ	3	122633050	4.48E-06	
rs6500842	16	6808287	0.541	0.87	ILMN_1716400	FOXM1	12	2837461	4.74E-06	
rs7205424	16	6805109	0.255	0.531	ILMN_1665737	UFD1L	22	17817951	4.87E-06	
rs6500842	16	6808287	0.541	0.87	ILMN_1675266	NAGPA	16	5015039	6.18E-06	
rs12445513	16	6843082	0.118	0.47	ILMN_1729734	MFSD4	1	203837106	6.27E-06	
rs6500842	16	6808287	0.541	0.87	ILMN_1810550	BAIAP3	16	1339189	6.49E-06	
rs6500842	16	6808287	0.541	0.87	ILMN_1803124	BIRC5	17	73733114	6.55E-06	
rs6500842	16	6808287	0.541	0.87	ILMN_1798654	MCM6	2	136314638	6.59E-06	
rs7205424	16	6805109	0.255	0.531	ILMN_1786065	UHRF1	19	4912680	6.96E-06	
rs7205424	16	6805109	0.255	0.531	ILMN_2395236	CHEK2	22	27420025	8.06E-06	
rs7205424	16	6805109	0.255	0.531	ILMN_1776490	C17orf53	17	39595293	8.27E-06	
rs7205424	16	6805109	0.255	0.531	ILMN_1719232	DGCR14	22	17502578	8.43E-06	
rs7205424	16	6805109	0.255	0.531	ILMN_1683450	CDCA5	11	64601683	9.00E-06	
rs8060556	16	6808512	0.215	1	ILMN_1771852	ZNF575	19	48731821	9.30E-06	

b) SNPs in the rs11155012 region

SNP	Chr	Pos	rsq	D'	probe	gene	g_chr	gene_pos	p_value	
rs911081	6	139171906	0.123	0.857	ILMN_2367157	CHRFAM7A	15	28459848	2.21E-08	*
rs9495127	6	138918259	0.129	1	ILMN_1760708	CRYBB2	22	23955472	2.30E-07	*
rs7742202	6	139115973	0.11	0.627	ILMN_1688218	RASGRP4	19	43602502	5.51E-07	*
rs9495127	6	138918259	0.129	1	ILMN_2323801	MOCS1	6	39981286	5.60E-07	*
rs6570277	6	139119982	0.119	0.641	ILMN_1688218	RASGRP4	19	43602502	6.50E-07	*
rs9495127	6	138918259	0.129	1	ILMN_1783185	SOX21	13	94160237	7.02E-07	*
rs9495127	6	138918259	0.129	1	ILMN_2353276	UPK3B	7	75978966	1.65E-06	*
rs9321678	6	139196016	0.147	1	ILMN_2367157	CHRFAM7A	15	28459848	1.80E-06	*
rs4321841	6	139118463	0.103	0.53	ILMN_1732198	UTS2	1	7836091	3.14E-06	
rs4321841	6	139118463	0.103	0.53	ILMN_1671818	UTS2	1	7833477	3.42E-06	
rs2698744	6	139227390	0.111	0.643	ILMN_2062381	LCOR	10	98708585	6.17E-06	
rs4538729	6	139117034	0.152	0.581	ILMN_1671818	UTS2	1	7833477	8.01E-06	

Supplementary Table 5: *Kaplan-Meier Plotter* survival analysis of statistically significant trans-eQTL genes associated with the rs6500843 and rs11155012 loci. The end point of the analysis is 10-year relapse-free survival, and the analysis was performed among cases treated with adjuvant chemotherapy (n=425), cases not treated with systemic therapy (n=1877) and all cases irrespective of treatment (n = 3455). To detect genes with potential interaction with treatment, tests for heterogeneity have been carried out between treated and untreated cases for each gene.

SNP	Gene	HR	95% C.I.			p-value	p(het)*
**Chemotherapy-treated cases (n = 425)**							
rs6500843	*MCM3*	1.43	0.99	-	2.06	0.058	*ns*
rs6500843	*CENPM*	2.04	1.27	-	3.30	0.0027	*ns*
rs6500843	*PKMYT1*	1.81	1.20	-	2.73	0.0044	*ns*
rs6500843	*AURKA*	1.25	0.89	-	1.77	0.2	0.0209
rs6500843	*KIFC1*	1.91	1.30	-	2.81	0.00077	*ns*
rs6500843	*TK1*	1.58	1.10	-	2.27	0.013	*ns*
rs11155012	*CHRFAM7A*	0.77	0.54	-	1.09	0.13	*ns*
rs11155012	*CRYBB2*	1.18	0.82	-	1.70	0.37	*ns*
rs11155012	*RASGRP4*	*na*		*na*		*na*	*n/a*
rs11155012	*MOCS1*	1.36	0.91	-	2.05	0.13	*ns*
rs11155012	*SOX21*	1.45	1.02	-	2.06	0.039	0.0239
**Cases without adjuvant chemotherapy (n = 1877)**							
rs6500843	*MCM3*	1.46	1.23	-	1.74	1.20E-05	*ns*
rs6500843	*CENPM*	1.91	1.61	-	2.27	5.90E-14	*ns*
rs6500843	*PKMYT1*	1.65	1.38	-	1.96	2.10E-08	*ns*
rs6500843	*AURKA*	2.34	1.91	-	2.88	0.00E+00	0.0209
rs6500843	*KIFC1*	2.02	1.70	-	2,4	3.30E-16	*ns*
rs6500843	*TK1*	1.79	1.50	-	2.12	1.90E-11	*ns*
rs11155012	*CHRFAM7A*	0.85	0.70	-	1.03	0.093	*ns*
rs11155012	*CRYBB2*	0.79	0.67	-	0.95	0.0097	*ns*
rs11155012	*RASGRP4*	*na*		*na*		*na*	*n/a*
rs11155012	*MOCS1*	0.79	0.66	-	0.94	0.0089	*ns*
rs11155012	*SOX21*	0.82	0.69	-	0.98	0.029	0.0239
**All cases (n = 3455)**							
rs6500843	*MCM3*	1.31	1.16	-	1.49	2.60E-05	-
rs6500843	*CENPM*	1.72	1.50	-	1.98	6.80E-15	-
rs6500843	*PKMYT1*	1.34	1.18	-	1.52	5.90E-06	-
rs6500843	*AURKA*	2.44	2.06	-	2.89	0	-
rs6500843	*KIFC1*	1.69	1.49	-	1.91	0	-
rs6500843	*TK1*	1.55	1.38	-	1.75	7.10E-13	-
rs11155012	*CHRFAM7A*	0.85	0.73	-	0.97	0.018	-
rs11155012	*CRYBB2*	0.66	0.58	-	0.75	2.50E-11	-
rs11155012	*RASGRP4*	*na*		*na*		*na*	-
rs11155012	*MOCS1*	0.61	0.54	-	0.69	8.90E-16	-
rs11155012	*SOX21*	0.62	0.55	-	0.70	3.40E-14	-

* Z-test for heterogeneity between treated and untreated, BH-adjusted for multiple testing

Supplementary Table 6: Summary of the HaploReg analysis results for the rs11155012 locus. The rs11155012 SNP is shown in yellow. Linked promoter (green) and enhancer (orange) sites have been marked in the table.

rsID	chr	pos	r2	D'	Promoter_ENCODE	Enhancer_ENCODE	DNAse_evidence	Protein_binding_evidence	Binding_motif_evidence	GENCODE_name
rs13209413	6	139050249	0.2	0.5	.	.	.	.	.	RP11-390P2.4
rs10457675	6	139050948	0.2	0.51	.	.	.	.	Yes	RP11-390P2.4
rs10457676	6	139051550	0.2	0.51	.	.	.	.	Yes	RP11-390P2.4
rs5880399	6	139054508	0.2	0.52	.	.	.	.	Yes	RP11-390P2.4
rs6938663	6	139055240	0.2	0.52	.	.	.	.	Yes	RP11-390P2.4
rs10457677	6	139055546	0.2	0.52	.	.	.	.	Yes	RP11-390P2.4
rs12206431	6	139056090	0.2	0.52	.	.	.	.	Yes	RP11-390P2.4
rs12194617	6	139063878	0.3	0.53	.	.	.	.	Yes	CCDC28A
rs12200534	6	139069323	0.2	0.52	.	.	.	.	Yes	CCDC28A
rs139233693	6	139071974	0.2	0.64	.	.	.	.	Yes	CCDC28A
rs73774898	6	139072232	0.3	0.65	.	.	.	.	Yes	CCDC28A
rs12194832	6	139073407	0.3	0.66	.	.	Yes	Yes	Yes	CCDC28A
rs201825583	6	139089068	0.3	0.69	.	Huvec,7_Weak_Enhancer	.	.	Yes	CCDC28A
rs200050818	6	139089069	0.3	0.69	.	Huvec,7_Weak_Enhancer	.	.	Yes	CCDC28A
rs11154998	6	139089070	0.3	0.69	.	Huvec,7_Weak_Enhancer	.	.	Yes	CCDC28A
rs12210412	6	139091791	0.3	0.7	.	.	.	.	Yes	CCDC28A
rs11155000	6	139099401	0.3	0.7	.	.	.	.	Yes	CCDC28A
rs7753418	6	139104107	0.2	0.57	.	.	.	.	.	CCDC28A
rs6570281	6	139105519	0.2	0.53	.	.	.	.	Yes	CCDC28A
rs7772915	6	139105637	0.2	0.54	.	.	.	.	Yes	CCDC28A
rs7773593	6	139105923	0.2	0.54	.	.	.	.	Yes	CCDC28A
rs3763185	6	139106599	0.3	0.71	.	.	.	.	Yes	CCDC28A
rs3763186	6	139106722	0.2	0.54	.	.	.	.	Yes	CCDC28A
rs3763187	6	139106787	0.2	0.54	.	.	.	.	Yes	CCDC28A
rs7746303	6	139108026	0.2	0.54	.	.	.	.	Yes	CCDC28A
rs7751745	6	139109125	0.2	0.54	.	.	.	.	Yes	CCDC28A

rsID	chr	pos	r2	D'	Promoter_ENCODE	Enhancer_ENCODE	DNAse_evidence	Protein_binding_evidence	Binding_motif_evidence	GENCODE_name
rs2327900	6	139109784	0.2	0.54	.	.	.	.	Yes	CCDC28A
rs2327901	6	139110033	0.2	0.54	.	.	.	.	Yes	CCDC28A
rs11155003	6	139110345	0.2	0.54	.	.	.	.	.	CCDC28A
rs9495249	6	139110841	0.2	0.53	.	.	.	.	Yes	CCDC28A
rs9495252	6	139111372	0.2	0.54	.	.	.	.	Yes	CCDC28A
rs12205733	6	139112228	0.3	0.71	.	.	.	.	Yes	CCDC28A
rs7773485	6	139113098	0.2	0.58	.	.	.	.	Yes	CCDC28A
rs9321673	6	139113392	0.2	0.58	.	.	.	.	Yes	CCDC28A
rs34409315	6	139113636	0.2	0.58	.	.	.	.	Yes	CCDC28A
rs139961983	6	139113869	0.3	0.71	.	.	Yes	.	Yes	CCDC28A
rs201682274	6	139114731	0.2	0.57	.	.	.	.	Yes	CCDC28A
rs2327902	6	139114732	0.2	0.61	.	.	.	.	Yes	CCDC28A
rs5880402	6	139115297	0.2	0.57	.	.	.	.	Yes	CCDC28A
rs1474962	6	139115518	0.2	0.58	.	.	.	.	.	CCDC28A
rs1474963	6	139115882	0.2	0.58	.	.	.	.	Yes	ECT2L
rs12198968	6	139120627	0.5	0.75	.	.	.	.	Yes	ECT2L
rs79981479	6	139120787	0.5	0.77	.	.	.	.	Yes	ECT2L
rs10457678	6	139122240	0.5	0.77	.	.	Yes	Yes	.	ECT2L
rs9321674	6	139122341	0.5	0.77	.	.	.	.	Yes	ECT2L
rs12664876	6	139132840	0.4	0.73	.	.	.	.	Yes	ECT2L
rs9403003	6	139134041	0.3	0.58	.	.	.	.	Yes	ECT2L
rs746865	6	139134103	0.3	0.58	.	.	.	.	Yes	ECT2L
rs746864	6	139134112	0.3	0.58	.	.	.	.	Yes	ECT2L
rs4895548	6	139134754	0.2	-1	.	HepG2,4_Strong_Enhancer	.	.	Yes	ECT2L
rs1321356	6	139134978	0.2	-1	.	HepG2,4_Strong_Enhancer;HMEC,7_Weak_Enhancer;NHEK,7_Weak_Enhancer	.	.	.	ECT2L
rs9389619	6	139135077	0.7	0.97	.	HepG2,4_Strong_Enhancer;HMEC,7_Weak_Enhancer;NHEK,7_Weak_Enhancer	.	.	Yes	ECT2L

rsID	chr	pos	r2	D'	Promoter_ENCODE	Enhancer_ENCODE	DNAse_evidence	Protein_binding_evidence	Binding_motif_evidence	GENCODE_name
rs9403004	6	139135392	0.2	-0.9	.	HepG2,4_Strong_Enhancer;NHEK,7_Weak_Enhancer;Huvec,5_Strong_Enhancer;HMEC,6_Weak_Enhancer	Yes	Yes	Yes	ECT2L
rs9376372	6	139135990	0.2	-1	.	HepG2,4_Strong_Enhancer;NHEK,7_Weak_Enhancer;HMEC,7_Weak_Enhancer	.	.	Yes	ECT2L
rs11155008	6	139137500	1	0.99	.	NHEK,7_Weak_Enhancer;HMEC,5_Strong_Enhancer	Yes	Yes	Yes	ECT2L
rs11155009	6	139137778	1	1	.	HMEC,5_Strong_Enhancer;NHEK,5_Strong_Enhancer	.	.	Yes	ECT2L
rs4896407	6	139137868	0.2	-0.9	.	HMEC,5_Strong_Enhancer;NHEK,5_Strong_Enhancer	.	.	Yes	ECT2L
rs6570288	6	139139589	0.2	-1	.	Huvec,5_Strong_Enhancer;HepG2,6_Weak_Enhancer;GM12878,6_Weak_Enhancer;NHEK,4_Strong_Enhancer;HSMM,7_Weak_Enhancer;H1,7_Weak_Enhancer;HMEC,4_Strong_Enhancer	.	.	Yes	ECT2L
rs12055486	6	139140446	0.3	-1	.	Huvec,6_Weak_Enhancer;GM12878,6_Weak_Enhancer;HMEC,5_Strong_Enhancer;NHEK,5_Strong_Enhancer	Yes	.	Yes	ECT2L
rs35219429	6	139140679	1	1	.	Huvec,6_Weak_Enhancer;GM12878,6_Weak_Enhancer;HMEC,5_Strong_Enhancer;NHEK,5_Strong_Enhancer	Yes	.	Yes	ECT2L
rs6570290	6	139140891	0.6	0.98	.	NHEK,5_Strong_Enhancer;HMEC,7_Weak_Enhancer;GM12878,7_Weak_Enhancer	.	.	Yes	ECT2L
rs9321676	6	139141089	0.2	1	.	NHEK,5_Strong_Enhancer;GM12878,7_Weak_Enhancer;HMEC,5_Strong_Enhancer	.	.	Yes	ECT2L
rs9321677	6	139141137	0.2	1	.	NHEK,5_Strong_Enhancer;GM12878,7_Weak_Enhancer;HMEC,5_Strong_Enhancer;HSMM,7_Weak_Enhancer	.	.	Yes	ECT2L
rs9389621	6	139141801	0.3	-1	.	NHEK,5_Strong_Enhancer;GM12878,7_Weak_Enhancer;HMEC,5_Strong_Enhancer	.	.	Yes	ECT2L
rs11155010	6	139142400	0.6	0.99	HepG2,3_Poised_Promoter	GM12878,7_Weak_Enhancer;HMEC,5_Strong_Enhancer;NHEK,7_Weak_Enhancer	Yes	.	Yes	ECT2L

rsID	chr	pos	r2	D'	Promoter_ENCODE	Enhancer_ENCODE	DNAse_evidence	Protein_binding_evidence	Binding_motif_evidence	GENCODE_name
rs12193938	6	139142855	1	1	HepG2,2_Weak_Promoter	HMEC,7_Weak_Enhancer	Yes	Yes	Yes	ECT2L
rs12193993	6	139142909	1	1	HepG2,2_Weak_Promoter	.	Yes	Yes	Yes	ECT2L
rs998035	6	139142941	0.6	0.99	HepG2,2_Weak_Promoter	.	Yes	Yes	Yes	ECT2L
rs7773177	6	139143088	1	1	HepG2,2_Weak_Promoter	.	.	.	Yes	ECT2L
rs6570291	6	139143274	1	1	HepG2,2_Weak_Promoter	.	.	.	Yes	ECT2L
rs10872493	6	139147421	0.6	0.98	.	.	.	.	Yes	ECT2L
rs10872494	6	139148135	0.6	0.96	.	.	.	.	Yes	ECT2L
rs11155012	6	139151784	1	1	.	.	.	.	Yes	ECT2L
rs11155013	6	139151838	0.9	0.99	.	.	.	.	Yes	ECT2L
rs55747302	6	139153882	0.6	0.97	.	.	.	.	Yes	ECT2L
rs9389625	6	139154056	0.4	0.98	.	.	.	.	Yes	ECT2L
rs12205070	6	139154247	1	1	.	.	.	.	Yes	ECT2L
rs10872495	6	139155677	1	0.99	.	.	.	.	Yes	ECT2L
rs1529152	6	139155855	0.3	-1	.	.	Yes	.	Yes	ECT2L
rs9285487	6	139156267	0.3	-1	.	.	.	.	Yes	ECT2L
rs34665665	6	139156282	1	0.99	.	.	.	.	Yes	ECT2L
rs6916561	6	139157259	0.3	-1	.	.	.	.	Yes	ECT2L
rs12207788	6	139157314	1	0.99	.	.	.	.	Yes	ECT2L
rs71766787	6	139157807	0.3	-1	.	.	Yes	.	Yes	ECT2L
rs4896408	6	139158129	0.3	-1	.	.	Yes	.	Yes	ECT2L
rs4896409	6	139158192	0.3	-1	.	.	Yes	.	Yes	ECT2L
rs10872496	6	139158676	1	0.99	.	.	Yes	.	Yes	ECT2L
rs3886649	6	139158791	0.3	-1	.	.	.	.	Yes	ECT2L
rs2017360	6	139159206	0.3	-1	.	.	.	.	Yes	ECT2L
rs9321680	6	139159382	0.3	-1	.	.	.	.	Yes	ECT2L

rsID	chr	pos	r2	D'	Promoter_ENCODE	Enhancer_ENCODE	DNAse_evidence	Protein_binding_evidence	Binding_motif_evidence	GENCODE_name
rs9385842	6	139178400	0.3	0.66	.	.	.	.	Yes	ECT2L
rs6899686	6	139179061	0.3	0.67	.	.	Yes	Yes	Yes	ECT2L
rs11155014	6	139180152	0.3	0.65	.	.	.	.	Yes	ECT2L
rs10872497	6	139180200	0.3	0.65	.	.	.	.	Yes	U6
rs10872498	6	139180508	0.3	0.65	.	.	.	.	Yes	ECT2L
rs10457679	6	139180868	0.3	0.65	.	.	.	.	Yes	ECT2L
rs71754397	6	139181653	0.3	0.64	.	.	.	.	Yes	ECT2L
rs11155015	6	139182263	0.2	0.51	.	.	.	.	.	ECT2L
rs11155016	6	139182265	0.3	0.51	.	.	.	.	Yes	ECT2L
rs2038444	6	139182617	0.3	0.65	.	.	.	.	Yes	ECT2L
rs2038445	6	139182742	0.3	0.64	.	.	.	.	Yes	ECT2L
rs2038446	6	139182979	0.3	0.65	.	.	.	.	Yes	ECT2L
rs12204850	6	139183879	0.3	0.65	.	HSMM,7_Weak_Enhancer	Yes	.	Yes	ECT2L
rs12205077	6	139184362	0.3	0.65	.	HSMM,7_Weak_Enhancer	.	.	Yes	ECT2L
rs12205026	6	139184382	0.3	0.65	.	HSMM,7_Weak_Enhancer	Yes	.	Yes	ECT2L
rs4896412	6	139184478	0.3	0.65	.	HSMM,7_Weak_Enhancer	Yes	.	Yes	ECT2L
rs4896413	6	139184640	0.3	0.65	.	HSMM,6_Weak_Enhancer	Yes	Yes	Yes	ECT2L
rs12211862	6	139185884	0.3	0.65	.	HSMM,5_Strong_Enhancer	Yes	.	Yes	ECT2L
rs12198757	6	139186457	0.3	0.65	.	HSMM,7_Weak_Enhancer	.	.	Yes	ECT2L
rs12200274	6	139186751	0.3	0.65	.	.	.	.	.	ECT2L
rs12200387	6	139186961	0.3	0.65	.	.	.	.	Yes	ECT2L
rs12213988	6	139187231	0.3	0.63	.	.	.	.	Yes	ECT2L
rs12215577	6	139187656	0.3	0.65	.	.	.	.	Yes	ECT2L
rs12215681	6	139187866	0.3	0.65	.	.	.	.	Yes	ECT2L
rs10457680	6	139190959	0.3	0.65	.	NHEK,6_Weak_Enhancer;HMEC,7_Weak_Enhancer;GM12878,5_Strong_Enhancer	Yes	.	Yes	ECT2L
rs11155017	6	139192425	0.3	0.65	.	GM12878,7_Weak_Enhancer	.	.	.	ECT2L
rs9495277	6	139193041	0.3	0.65	.	GM12878,7_Weak_Enhancer	.	.	Yes	ECT2L
rs10457681	6	139193565	0.3	0.65	.	GM12878,7_Weak_Enhancer	Yes	.	Yes	ECT2L

rsID	chr	pos	r2	D'	Promoter_ENCODE	Enhancer_ENCODE	DNAse_evidence	Protein_binding_evidence	Binding_motif_evidence	GENCODE_name
rs10872499	6	139193603	0.3	0.65	.	GM12878,7_Weak_Enhancer	Yes	.	Yes	ECT2L
rs10872500	6	139193673	0.3	0.65	.	GM12878,7_Weak_Enhancer	.	.	Yes	ECT2L
rs9484210	6	139194792	0.3	0.65	.	.	.	.	Yes	ECT2L
rs1157388	6	139195283	0.3	0.64	.	.	Yes	.	Yes	ECT2L

Supplementary Table 7: Breast Cancer Association Consortium (BCAC) studies genotyped on the iCOGS chip and represented in this work.

Study Acronym	Study	Country	Cases[a]	Events[a]	Study design
ABCFS	Australian Breast Cancer Family Study	Australia	671	160	Cancer registries in Victoria and New South Wales (1992-1999): all cases from Melbourne and Sydney diagnosed before age 40 plus a random sample of those diagnosed at ages 40-59.
ABCS	Amsterdam Breast Cancer Study	Netherlands	443	60	Breast cancer patients diagnosed before age 50 in 2003-2009 at the NKI-AVL; and (ABCS-F) All non-BRCA1/2 breast cancer cases from the family cancer clinic of the NKI-AVL tested in the period 1995-2009; all ages and diagnosed with breast cancer in 1965-2008.
BBCC	Bavarian Breast Cancer Cases and Controls	Germany	248	16	Consecutive, unselected cases with invasive breast cancer recruited at the University Breast Centre, Franconia in Northern Bavaria from 2002-2010.
HEBCS	Helsinki Breast Cancer Study	Finland	1451	199	(1) Consecutive cases (883) from the Department of Oncology, Helsinki University Hospital 1997-8 and 2000, (2) Consecutive cases (986) from the Department of Surgery, Helsinki University Hospital 2001–2004, (3) Familial breast cancer patients (536) from the Helsinki University Hospital, Departments of Oncology and Clinical Genetics (1995-).
KARBAC	Karolinska Breast Cancer Study	Sweden	409	106	1. Familial cases from Department of Clinical Genetics, Karolinska University Hospital, Stockholm. 2. Consecutive cases from Department of Oncology, Huddinge & Söder Hospital, Stockholm 1998-2000.
KBCP	Kuopio Breast Cancer Project	Finland	410	154	Women seen at Kuopio University Hospital between 1990-1995 because of a breast lump, mammographic abnormality, or other breast symptom and who were found to have breast Cancer.
kConFab/AOCS	Kathleen Cuningham Foundation Consortium for Research into Familial Breast Cancer / Australian Ovarian Cancer Study	Australia	183	16	Index (youngest affected) cases from BRCA1-and BRCA2-mutation-negative multiple-case breast and breast-ovarian families recruited though family cancer clinics from across Australia and New Zealand from 1998-present.
LMBC	Leuven Multidisciplinary Breast Centre	Belgium	2074	67	All patients diagnosed with breast cancer and seen in the Multidisciplinary Breast Centre in Leuven (Gashuisberg) since June 2007 plus retrospective collection of cases diagnosed since 2000.
MARIE	Mammary Carcinoma Risk Factor Investigation	Germany	1635	244	Incident cases diagnosed from 2001-2005 in the study region Hamburg in Northern Germany, and from 2002-2005 in the study region Rhein-Neckar-Karlsruhe in Southern Germany.

Study Acronym	Study	Country	Cases[a]	Events[a]	Study design
MCBCS	Mayo Clinic Breast Cancer Study	USA	1198	100	Incident cases residing in 6 states (MN, WI, IA, IL, ND, SD) seen at the Mayo Clinic in Rochester, MN from 2002-5
OFBCR	Ontario Familial Breast Cancer Registry	Canada	686	80	Invasive cases aged 20-54 years identified from the Ontario Cancer Registry from 1996-1998. All those at high genetic risk were eligible; random samples of women not meeting these criteria were also asked to participate.
PBCS	NCI Polish Breast Cancer Study	Poland	389	35	Incident cases from 2000-2003 identified through a rapid identification system in participating hospitals covering ~ 90% of all eligible cases, and cancer registries in Warsaw and Łódź covering 100% of all eligible cases.
RBCS	Rotterdam Breast Cancer Study	Netherlands	529	84	Familial breast cancer patients selected from the clinical genetics centre at Erasmus Medical Centre between 1994-2005.
SASBAC	Singapore and Sweden Breast Cancer Study	Sweden	1146	155	Women diagnosed in Sweden aged 50-74 in 1993-1995.
SEARCH	Study of Epidemiology & Risk Factors in Cancer Heredity	UK	6233	721	Identified through the Eastern Cancer Registration and Information Centre: (I) prevalent Cases; diagnosed 1991-1996; under 55 years of age at diagnosis; recruited 1996-2002 (ii) incidence cases; diagnosed since 1996; under 70 years of age at diagnosis; recruited 1996-present.
SKKDKFZS[b]	Städtisches Klinikum Karlsruhe Deutsches Krebsforschungszentrum Study	Germany	123	21	Women diagnosed with primary in situ or invasive breast cancer at the Städtisches Klinikum Karlsruhe from March 1993 to July 2005. Cases were 21-93 years of age.

[a] Counts indicate cases eligible for the current study (treatment and follow-up information available, follow-up time > recruitment latency).
[b] While SKKDKFZS is a BCAC study, this data set was genotyped as part of the Triple Negative Consortium (TNBCC).
